# Are you afraid of the dark? Notes on the psychology of belief in histories of science and the occult[Atl-fn FN0001]

**DOI:** 10.1080/13642537.2016.1170062

**Published:** 2016-04-15

**Authors:** Andreas Sommer

**Affiliations:** ^a^ Churchill College & Department of History and Philosophy of Science, University of Cambridge, Cambridge, UK

**Keywords:** Historiography, history of science, parapsychology, psychical research, pragmatism, psychology of belief, Historiografie, Wissenschaftsgeschichte, Parapsychologie, Erforschung von Übersinnlichem, Pragmatismus, Psychologie des Glaubens, historiografía, historia de la ciencia, parapsicología, investigación psíquica, pragmatismo, psicología de la creencia, storiografia, storia della scienza, parapsicologia, ricerca psicologica, pragmatismo, psicologia delle credenze, historiographie, histoire des sciences, parapsychologie, recherche psychique, pragmatisme, psychologie de la croyance, ιστοριογραφία, ιστορία των επιστημών, παραψυχολογία, ψυχική έρευνα, πραγματισμός, ψυχολογία της πίστης

## Abstract

The popular view of the inherent conflict between science and the occult has been rendered obsolete by recent advances in the history of science. Yet, these historiographical revisions have gone unnoticed in the public understanding of science and public education at large. Particularly, reconstructions of the formation of modern psychology and its links to psychical research can show that the standard view of the latter as motivated by metaphysical bias fails to stand up to scrutiny. After highlighting certain basic methodological maxims shared by psychotherapists and historians, I will try to counterbalance simplistic claims of a ‘need to believe’ as a precondition of scientific open-mindedness regarding the occurrence of parapsychological phenomena by discussing instances revealing a presumably widespread ‘will to disbelieve’ in the occult. I shall argue that generalized psychological explanations are only helpful in our understanding of history if we apply them in a symmetrical manner.

## Abstract (de)

Infolge der neuesten Fortschritte innerhalb der Wissenschaftsgeschichte gilt der bis dato gängige Blick auf den inhärenten Konflikt zwischen Wissenschaft und dem Okkulten als hinfällig. Diese Neuerungen innerhalb der Wissenschaft wurden jedoch von der Öffentlichkeit nicht wahrgenommen. Insbesondere die Rekonstruktion der Genese der modernen Psychologie und ihren Verbindungen zu parapsychologischen Forschungen zeigen, dass sich letztere nicht einfach als Ausdruck von metaphysischer Voreingenommenheit verstehen lassen. Um nun einen Ausgleich zu schaffen, werde ich Fälle eines ,‘Willens zum Unglauben‘ diskutieren und davon ausgehend den Vorschlag machen, dass allgemeine psychologische Erklärungen nur dann hilfreich für ein Geschichtsverständnis sind, wenn wir sie in einer symmetrischen Art und Weise anwenden.

## Abstract (es)

La visión popular del conflicto inherente entre la ciencia y lo oculto, ha quedado obsoleta debido a los avances recientes en la historia de la ciencia. Sin embargo, estas revisiones historiográficas han pasado casi desapercibidas en la comprensión pública de la ciencia y la educación. Particularmente las reconstrucciones de la formación de la psicología moderna y sus conexiones con la investigación psíquica, nos muestran que la visión común de esta última, motivada por sesgos metafísicos no pasa la prueba de la realidad. En este artículo discuto ejemplos de una ‘’voluntad de incredulidad’’ para contrabalancear las demandas simplistas de una ‘’necesidad de creer’’ como precondición de una apertura científica de la mente en relación con la incidencia de fenómenos parapsicológicos, y sugiero que las explicaciones psicológicas generalizadas son útiles solamente en nuestra comprensión de la historia si las aplicamos de manera simétrica.

## Abstract (it)

La rappresentazione popolare della contrapposizione tra scienza e occulto è stata resa obsoleta dai recenti progressi della storia della scienza. Eppure, queste revisioni storiografiche sono state trascurate dalla conoscenza comune sulla scienza e dall'istruzione pubblica in generale. In particolare la ricostruzione di come si sia formata la psicologia moderna e dei suoi collegamenti con la ricerca psicologica dimostra una visione standardizzata di questi ultimi, giustificata da pregiudizi metafisici che non reggono la verifica. Per controbilanciare affermazioni semplicistiche relative a un 'bisogno di credere' come precondizione di una scientifica apertura mentale relativa all'insorgenza di fenomeni parapsicologici, discuto le istanze della 'volontà di credere' e suggeriscono che le spiegazioni psicologiche generiche sono utili alla nostra comprensione della storia solamente se le usiamo in modo bilanciato.

## Abstract (fr)

L’opinion populaire concernant le conflit intrinsèque entre la science et l’occulte a été rendue obsolète par les récentes avancées de l’histoire des sciences. Pourtant ces révisions historiographiques sont passées inaperçues de la compréhension publique de la science et de l’éducation publique dans son ensemble. Les reconstructions de l’évolution de la psychologie moderne et de ses liens avec la recherche psychique peuvent montrer en particulier que la vision standard de cette dernière en tant que motivée par un bais métaphysique échoue à résister à son examen. Pour contrebalancer l’argument simpliste d’un ‘besoin de croire’ comme précondition à l’ouverture d’esprit scientifique concernant l’occurrence des phénomènes parapsychologiques, des exemples sont ici discutés de ‘volonté de ne pas croire’ et il est suggéré que des explications psychologiques généralisées sont seulement utiles pour nous aider à comprendre l’histoire à condition de les appliquer de manière symétrique.

## Abstract (el)

Η δημοφιλής άποψη της εγγενούς σύγκρουσης μεταξύ της επιστήμης και του μεταφυσικού έχει καταστεί παρωχημένη από τις πρόσφατες εξελίξεις στην ιστορία της επιστήμης. Ωστόσο, αυτές οι ιστοριογραφικές αναθεωρήσεις έχουν περάσει απαρατήρητες στη δημόσια κατανόηση της επιστήμης και της δημόσιας εκπαίδευσης γενικότερα. Οι ιδιαίτερες ανακατασκευές της διαμόρφωσης της σύγχρονης ψυχολογίας και η σύνδεσή της με την ψυχική έρευνα μπορούν να καταδείξουν ότι η κανονική οπτική της τελευταίας ως κινούμενης από την μεταφυσική προκατάληψη αποτυγχάνει να αντέξει τον λεπτομερή έλεγχο. Προκειμένου να αντισταθμίσω απλοϊκές αξιώσεις μιας «ανάγκης να πιστέψουμε» ως προϋπόθεση της επιστημονικής ευρύτητας του πνεύματος όσον αφορά την εμφάνιση των παραψυχολογικών φαινομένων, θα συζητήσω τις περιπτώσεις της «θέλησης να δυσπιστήσουμε» και δείχνω ότι οι γενικευμένες ψυχολογικές εξηγήσεις είναι χρήσιμες μόνο στην κατανόηση της ιστορίας αν εφαρμόζονται με συμμετρικό τρόπο.

## ‘Ways of being in the world’ in historical research and the therapeutic setting

At first glance, psychotherapists and historians appear to have very little in common. To be sure, both professions are concerned with human beings, but your clients are obviously alive, while my historical protagonists are long gone. The persons you work with usually seek you out to get help understanding and changing their individual present, whereas I select my historical actors in the hope they might prove useful to me as a lens to understand collective pasts. You empower your clients to become active collaborators in the therapeutic process by encouraging them to mobilize own resources, while my historical actors are perfectly at my mercy should I chose to distort their lives to make them fit any preconceived narratives of mine. Not least, your clients are protected by basic human rights and can take legal steps if they feel mistreated, whereas I have nothing to fear in consequence of, say, retroactively tainting a historical protagonist’s reputation as the dead are unable to sue.

Yet, it seems that in a crucial sense some of these differences actually indicate a mutual work ethos. I take it for granted that the first step in establishing a fruitful client-therapist relationship requires the therapist’s commitment to treat those seeking help on their own terms. Rather than forcing your own way of being in the world upon persons in your care, you will strive to base therapeutic interventions on a thorough understanding of where each is coming from. Ideally, historians are trained to observe very similar methodological maxims. For our job is no longer to justify the present by limiting reconstructions of the past through compatibilities with today’s epistemological and metaphysical standards, but to faithfully resurrect the past by doing our best to obtain a thorough understanding of sentiments and existential categories that were actually at the disposal of the individuals whose ways of being in the world we aim to investigate.

Quite often, I struggle to get my head around beliefs and sentiments of my historical actors, even if I know this doesn’t necessarily require me to drastically modify my own presuppositions and cultural conditionings. I expect similar issues to arise as challenges to therapeutic practice. A client may, for instance, report a certain class of recurring ‘weird’ experiences, such as fulfilled prophetic dreams of accidents and deaths, possibly intrusive telepathic rapport with a parent or lover they are in the process of separating from, frightening out-of-body experiences, visual or auditory hallucinations of dead relatives and friends, or dramatic ‘poltergeist’-style episodes involving loud noises, levitating objects, and other ‘things that go bump’ in their homes or maybe even workplaces. In many cases, you may find it advisable not to encourage your client’s belief in the reality of the reported phenomena, while trying to establish what emotional conflicts and issues each experience may represent.

On the other hand, you might have encountered instances where ostensibly ‘paranormal’ experiences, rather than being inherently unsettling, on the contrary inspired a client’s confidence in higher and ultimately benevolent realities. Far from persuading such clients to abandon these apparently irrational and naive beliefs, you may have come to acknowledge that at least some individuals can exploit profound forms of ‘transpersonal’ optimism as highly effective means to cope with, and possibly even overcome, concrete hardships and emotional problems. And from conversations with various therapists I’m practically certain that there are cases where a client’s fear of being considered ‘not normal’ or mentally ill simply by virtue of having such experiences constitute a major obstacle to therapeutic progress. After all, most of us were brought up in the belief that science has conclusively shown that these things are impossible, and that something must be wrong with those reporting experiences that appear to suggest otherwise.

Obviously, as a historian, I have no intention let alone competence to argue for the existence or non-existence of parapsychological (or ‘psi’) phenomena. It is merely as a potential token of assistance with such cases – however small it will be – the present article is written. In a sense, it could be viewed as complementary to recent clinical studies and revisions appearing to show that, whatever their ultimate nature, exceptional or ‘paranormal’ experiences are neither particularly uncommon nor intrinsically pathological (cf. Cardeña, Lynn, & Krippner, 2014). In fact, some of the recent historical research I shall try to distil in the following pages has revealed that the ‘occult’ was always a part of our scientific and intellectual heritage.

## Science as a candle in the dark?

Unless you have had striking experiences of a seemingly occult nature yourself, you’re probably not likely to believe that ‘psi’ phenomena occur. But even if you do, you probably know that it is wise to keep that belief to yourself if you expect your peers to view you as sane, critical and scientifically minded. And supposing you’re a sceptic, you demand that belief should depend on sound empirical evidence, because the more outlandish a proposition the stronger the evidence must be to support it. But there simply is no scientific evidence, because wouldn’t we all know if there was? For science, we have been brought up to believe, is intrinsically self-correcting and always on the lookout for anomalies that might bring about revolutionary scientific breakthroughs. Moreover, the very essence of scientific practice securing its self-correcting nature are intellectual core virtues – impartial love of truth, open-mindedness paired with discerning rigour, courageous anti-dogmatism and other qualities without which the scientific enterprise would quickly lose its appeal as intrinsically progressive and good.

Those holding this quasi-teleological view of scientific progress are also likely to believe the study of the history of science and medicine is irrelevant: if science always provides the most reliable mirror of reality, its past can constitute little more than a graveyard of errors and obsolete ideas. For many, the only story worthwhile telling is in the style of that modern bible of popular science, Carl Sagan’s *The Demon*-*Haunted World: Science as Candle in the Dark* (Sagan, 1995). In fact, subsequent celebrity scientists with a metaphysical axe to grind like Neil deGrasse Tyson and Bill Nye in the US, and Richard Dawkins and Brian Cox here in Britain, have closely adhered to this standard way of preaching to the masses the gospel of science as a grand master narrative of humanity’s journey from the deplorable, oppressive superstitions of the past towards the inherently liberating and humanistic (Western) sciences of the present.

Science popularizers laudably hammer home the message that science deserves that name only if it is firmly rooted in the intellectual virtues mentioned above, and if it strictly builds on the best available evidence. Curiously, however, these basic principles – which obviously should guide historical research no less than science – are nearly always dropped as soon as the question of the relationship between science and religion (the supposed breeding ground of occult belief) is concerned. Instead of systematic, impartial research, we find claims of their perennial incompatibility endlessly recycled in the mass media and the ‘public understanding of science’, while academic historical scholarship showing that the so-called conflict thesis of science and religion is largely a historiographical artefact stemming from the nineteenth century is simply ignored.

In fact, the popular notion of the supposedly self-evident opposition of science and religion – each routinely portrayed as monolithic entities epitomizing eternally progressive vs. regressive mindsets – turns out to be little more than a caricature, as soon as their interactions are reconstructed within original contexts and by paying attention to local, political, ideological and other factors usually passed over in triumphalist chronologies of progress (see, e.g. Brooke, 1991; Dixon, Cantor, & Pumfrey, 2010; Numbers, 2009).^1^ Like any other human endeavour, science is not practiced in a cultural, political and metaphysical vacuum, and it is these ‘extra-scientific’ conditions of the past that have profoundly shaped scientific institutions, methods, research questions, and theories up the present. Recent studies in the history of neuroscience, for example, have revealed that contrary to present-day popular beliefs, epiphemonenalist standard views are no unequivocal corollary of neuroscientific advances. The view that the brain produces the mind has always been just one among various pre-existing metaphysical presuppositions, for which the modern mind and brain sciences have served as vehicles (Hagner, 1992, 2012; Harrington, 1987; Smith, 1992; Vidal, 2009; Weidman, 1999; Young, 1970).

A related myth is the view of the inherent opposition of scientific psychology and the occult. Contrary to ongoing attempts to demarcate modern psychology from parapsychology through simplistic historical assertions of the latter’s intrinsic unscientificity (e.g. Ash, Gundlach, & Sturm, 2010; Marshall & Wendt, 1980), a clear-cut distinction has been difficult if not impossible to draw in terms of research, methods and representatives. This is particularly true for the infancy of professionalized psychology: Between 1889 and about 1909, investigations into ‘marvellous’ phenomena associated with mesmerism and spiritualism were discussed on important platforms of early academic psychology like the International Congresses of Psychology, which were initiated and organized by parapsychological researchers such as Charles Richet, Julian Ochorowicz, Arthur T. and Frederic W. H. Myers, Henry and Eleanor M. Sidgwick, and Albert von Schrenck-Notzing. ‘Founding fathers’ of the psychological profession, such as William James in the US and Théodore Flournoy in Switzerland, were active psychical researchers and attempted an integration of radical empirical parapsychological studies into fledgling psychology, while others, such as Théodule Ribot in France, appeared supportive of such attempts (Brower, 2010; Le Maléfan & Sommer, 2015; Plas, 2012; Shamdasani, 1994; Sommer, 2013a, 2013b; Taylor, 1985). Also flying in the face of assertions that scientific psychology had done away with the occult is the continuity of open-minded scientific interest in parapsychological phenomena within and beyond the psychological profession (Mauskopf & McVaugh, 1980; Sommer, 2013a, 2014b; Valentine, 2012).

## Wills to believe

*He who believes in it carries out experiments in sorcery, and he who does not believe in it as a rule does not. But since man is known to have a great tendency to find confirmed what he believes in, and to this end might even apply a great ingenuity to deceive himself, to me the success of such experiments only proves that those conducting them believe in them to begin with*. (Wundt, 1892, pp. 9–10)^2^

*The true opposites of belief, psychologically considered, are doubt and inquiry, not disbelief*. (James, 1889, p. 322)

To say that the occult entanglements of modern psychology, and the sciences in general, have been squarely written out of public and disciplinary history is certainly not an overly melodramatic statement.^3^ Interestingly, an axiom underlying the traditional historiography of science and the occult that has been obscuringing these links boils down to a psychological rather than historical explanation of open-minded scientific interest in occult phenomena, and a surprisingly simplistic one at that: metaphysical bias and an infantile ‘need to believe’ in transcendental realities.

The above quote by Wilhelm Wundt, the ‘father’ of professionalized psychology in Germany, shows that generalizing psychological explanations for scientific interest in ‘paranormal’ phenomena by an unhealthy obsession with the marvellous are not exactly new. In the US, Joseph Jastrow had launched his long career as self-appointed border-guard and popularizer of American psychology by proclaiming that open-minded scientific tests of the reported phenomena of spiritualism indicated a ‘state of mind that is to be prevented’ since it was ‘dangerous to mental sanity’ and ‘morbidly hungry for something unusual, something mystic, something occult’ (Jastrow, 1887, p. 8). A refusal to dismiss the occult was so dangerous for Jastrow and other opponents of psychical research ‘because this system goes deeper, and appeals to the feelings, that it blinds its adherents to sense and reasoning’ (loc. cit.). Much later, Edwin Boring, the eminent historian of experimental psychology, likewise insisted that it was ‘quite clear that interest in parapsychology has been maintained by faith. People want to believe in an occult something’ (Boring, 1966, p. xvi).

Unproblematic as such statements may seem at first glance, unfortunately the matter is not quite as straightforward. For once, we cannot simply assume that the remarkable outrage expressed by critics like Wundt, Jastrow and other hardliners in the fight against the ‘occult’ during the making of modern psychology was scientifically justified. Again and again, writing in their function as scientists, these critics in fact mainly relied on appeals to assumed social, cultural and not least religious dangers of a belief in ‘occult’ phenomena. Eschewing constructive dialogues with their targets of attack, opponents offered little dispassionate and constructive methodological critiques and favoured popular magazines and pamphlets rather than formal scholarly channels to get their polemics across. Epistemological positions, methods, aims and arguments of psychical researchers were misrepresented by reliance on generalized allegations of fraud and insinuations of methodological incompetence, the latter being tacitly explained through claims of metaphysical bias (Sommer, 2012, 2013a, Chapter 4; Taylor, 1996).

Moreover, by the late nineteenth century, any empirical approach to marvellous events had already been repudiated from intellectual discourse for over a century. Again contrary to widespread assumptions, however, it was predominantly political, philosophical and religious concerns rather than scientific work that had made fashionable the Enlightenment notion of belief in preternatural occurrences as an indicator of intellectual, moral and spiritual vulgarity at best, and mental illness at worst (cf. Cameron, 2010; Daston & Park, 1998; Porter, 1999; Sommer, 2013a, Chapter 1). Later, popularizers of professional psychology merely continued an overwhelmingly polemical war, relying on an Enlightenment standard rhetoric using fuzzy but immensely loaded terms such as ‘mysticism’, ‘superstition’, ‘sorcery’, ‘enthusiasm’ and similar catchwords to discredit intellectual interest in alleged occult phenomena. This strategy served to construct a public image of the ‘new psychology’ particularly in the US and Germany as inherently progressive and unified, and not least as practically useful in the combat of the supposed social and cultural dangers of spiritualism and other ‘epidemic delusions’ (Coon, 1992; Leary, 1987; Sommer, 2012).

Another stubborn myth regarding psychical research is that it has always been a reactionary movement, owing its existence to a childish reluctance to accept the self-evident truth of scientific materialism. While the history of scientific materialism itself thoroughly refutes the teleological standard narrative of materialism as a science-based and therefore obligatory worldview (Gregory, 1977; Lange, 1876–1877), not a few leading representatives of psychical research like its doyens in France (Charles Richet), Germany (Albert von Schrenck-Notzing), Poland (Julian Ochorowicz) and Italy (Enrico Morselli and Cesare Lombroso) have either been card-carrying materialists or positivists advocating a distinctively secular and anti-spiritualist psychical research (Brancaccio, 2014; Sommer, 2013a, Chapter 2, 2014a). To complicate matters further, we would be hard pressed to identify a single representative of scientific materialism among the early vocal psychological opponents of psychical research (cf. Hatfield, 1995). Not least, a continued openness to extra-sensory perception (ESP) within a distinctively materialist tradition, Freudian psychoanalysis (Devereux, 1974; Gyimesi, 2012; Totton, 2003), should make us sceptical of the psychical research vs. materialism stereotype. Yet, unchecked simplistic arguments from metaphysical bias that fail to stand up to historical scrutiny continue to be advanced even in professional philosophical discussions of parapsychology and the demarcation problem (Sommer, 2014a).

To simplify an immensely complicated story: the professionalization and beginning secularization of the sciences in the late nineteenth century occurred in an atmosphere that was marked by a vehement hostility not so much to religion but to ‘magical thinking’. Scientific secularization and the rise of positivism were driven not by a materialist worldview, but mainly by rationalist and predominantly anti-clerical religious thinkers, who more often than not were just as programmatically opposed to materialism as they were to spiritualism and related large-scale occult movements of the time.

The opposition to magical thinking also crystallized in rather dramatic political events. The birth of modern experimental psychology in Germany, for example, occurred at the end of Bismarck’s *Kulturkampf*. This was a national war against the Catholic church fought throughout the 1870s, which, after the March Revolution in 1848, could be called the German version of the French Revolution. Propagating an Enlightenment-style anti-‘superstition’ rhetoric, the *Kulturkampf* was vocally supported by leading popularizers of secularized science such as the outspoken anti-materialist Rudolf Virchow and the materialist Ernst Haeckel, who were both strictly unsympathetic to a radical empirical approach to the phenomena of spiritualism and mesmerism. The crucial thing to understand here is that opposition to investigations of the phenomena of mesmerism and spiritualism came from multiple and often mutually antagonistic camps. To say this was a climate not exactly conducive to parapsychological experimentation would therefore be an understatement.

When viewed in its original context, the aggressive opposition by early psychologists such as Wundt and Jastrow to unorthodox scientific activities appears to make sense in terms of a strategic imperative to protect the public image of nascent psychology from dangerous associations with the occult. The strange story of the coinage of the term ‘Parapsychologie’ by Max Dessoir also lends itself to an interpretation along these lines. Following attacks by Wundt and other leaders of the new psychological profession, Dessoir, a young psychologist who had initially tried to expand the methodological scope of German psychological experimentation in the late 1880s through an integration of parapsychological research, promptly embarked on a much safer career as a self-appointed guardian of rationality and *Volksaufklärer* (Sommer, 2013b).

But does political calculus and career opportunism really suffice to account for the ongoing bias in the public historiography of science and the occult? Although instances of violent opposition to new ideas is a commonplace in the history even of orthodox sciences, I cannot help but being struck by the persistent vehemence, the often hateful and emotional nature of some of the attacks that continue to inform this historiography.^4^ I find myself essentially in agreement with psychoanalyst William Gillespie and many others, who observed that there was a strong tendency among critics to respond to the data of psychical research ‘in an irrational, emotionally determined way’ (Gillespie, 1956, p. 209). In fact, while sweepingly accusing elite psychical researchers of a regressive and undisciplined ‘will to believe’, critics have at the same time displayed strong indications of various fears. The American neurologist George M. Beard, for example, was not exactly a model of a rational and calm response to spiritualism and its impartial investigation, when he recommended that for ‘logical, well-trained, truth-loving minds, the only security against spiritism is in hiding or running away’ (Beard, 1879, p. 73). When Wundt was challenged to justify his dismissal of the experimental evidence presented by eminent German physicists in support of the reality of some of the phenomena of spiritualism, his fears of a downfall of modern culture and religion following in the train of a radical empiricism apparently got the better of his scientific curiosity, for he proclaimed:

The moral barbarism produced in its time by the belief in witchcraft would have been precisely the same, if there had been real witches. *We can therefore leave the question entirely alone, whether or not you have ground to believe in the spiritualistic phenomena.* (Wundt, 1879, p. 592, my italics)

In France, the physicist Léon Foucault opposed investigations of table moving by exclaiming:

If I saw a straw moved by the action of my will … I should be terrified. If the influence of mind upon matter does not cease at the surface of the skin, there is no safety left in the world for anyone (quoted in Sudre, 1960, p. 33).

Now I don’t want to appear as trying to substitute one crude psychological explanation (‘interest in occult phenomena has been motivated by an irrational need to believe’, etc.) with another, equally simplistic one (‘opposition to psychical research has been motivated by irrational fears’) and use it as a historiographical argument. At the same time, once we acknowledge that cultural and personal biases constitute fundamental problems in any realm of human activity, the insight that we have to deal with them somehow seems inescapable. In the philosophy of science, the problem of incommensurability as formulated by writers like Thomas Kuhn and Paul Feyerabend already boils down to a squarely psychological one. Kuhn’s own thoughts on instances of dogmatism throughout the history of science, for example, cautiously drew on psychological experiments in cognitive dissonance (Kuhn, 1996, esp. pp. 63–65, 112–115 and Chapter 10). Kuhn’s ideas were also informed by the notion of ‘absolute presuppositions’ as discussed by the philosopher Robin Collingwood (1948). In the Kuhnian sense, these are fundamental propositions which scientists cannot afford to question or investigate but simply have to take for granted, such as the concept of causality, and the very possibility to get at fundamental truth in the first place.

This of course is the rationalist’s arch dilemma, which we also find at the heart of the pragmatist conception of truth. After stating that some of our most fundamental knowledge comes second hand and from unquestioned authorities, William James observed:

Our belief in truth itself, for instance, that there is a truth, and that our minds and it are made for each other, – what is it but a passionate affirmation of desire, in which our social system backs us up? (James, 1897, p. 9).

For James, a radical empirical psychology of belief was forced to acknowledge the tautological or self-confirming nature and foundation of much supposedly rational belief. In the final analysis, it was passion rather than reason that James found decided metaphysical positions and their rationalizations: Like anybody else, the philosopher consciously or unconsciously wants to be the world a certain way. It was his inevitable will to believe that

loads the evidence for him one way or the other, making for a more sentimental or a more hard-hearted view of the universe, just as this fact or that principle would. He *trusts* his temperament. Wanting a universe that suits it, he believes in any representation of the universe that does suit it (James, 1907, p. 7, original emphasis).

With James I should concede that a compartmentalization of mentalities into ‘tough-minded’ or rational vs. ‘tender-minded’ or sentimental ways of being in the world (or, as I would like to suggest adding, ‘Platonic’ vs. ‘Epicurean’) is ‘indeed monstrously over-simplified and rude’ (James, 1907, p. 35). But if we grant a near infinite variability of mixtures existing between these temperamental poles, it might serve some analytical purpose after all – particularly, if we are to get at possible reasons for the immense public appeal of the indefinitely more monstrously crude stereotypes regarding science, religion and the occult.

Indeed, James’ reflections on the inevitably irrational origins of belief may be as radical as Léon Foucault’s above expression of horror in the face of a psychokinetically moved straw is consequent. Superficially perceived, Foucault’s quote may have a paranoid or comical ring to it. But I think philosopher Stephen Braude has a point when he maintains that ‘it’s a very small step conceptually from psychokinetically nudging a matchstick to psychokinetically causing someone to drop dead, or causing a car to crash’ (Braude, 2007, p. 30).^5^ Such fears, according to Braude, might go a long way accounting for the often emotional off-hand dismissal of empirical indications in support of psi phenomena. Moreover, a psychoanalytic truism has it that most of us simply don’t want to know the innermost contents of our minds. If this is the case, how likely are we to welcome the prospect of others potentially having access? There might be good reasons why psi researchers have not only considered the fear of psi as a political problem, but also occasionally addressed it as a methodological issue (e.g. LeShan, 1966; Tart, 1984; Tart & LaBore, 1986).

Lastly, the ‘will to disbelieve’ in magical powers and correspondences may well be as old as the will to believe in them (cf. Whitmarsh, 2016). Philosopher Michael Grosso (1990, pp. 244–246) reminds us that the ancient Greek materialist philosophers Epicurus and Lucretius have been revered like messiahs by their disciples for liberating them of the fear of evil magic, capricious gods and spirits, and not least the horrifying uncertainty regarding the very nature of a hypothetical afterlife. Epiphenomenalism has always been a radical and convenient way to shut out these deep-seated existential fears, and to hold with authors like Otto Rank and Ernest Becker that the human desire for immortality was universal faces various problems. For once, Grosso argues that anthropologically and historically considered, the fear of death appears to be a relatively recent scourge of humankind, and might in fact be a main characteristic of modernity. With the anthropology of Sir James Frazer, Grosso also makes the interesting claim that fear *of the dead* is a much more promising universal than the fear of annihilation.^6^

At least a conscious antipathy towards the notion of immortality seems in fact fairly common. This has been suggested by the results of a survey on attitudes to immortality conducted by James’ fellow pragmatist and psychical researcher, F.C.S. Schiller (1904). The philosopher Bernard Williams (Williams, 1976, Chapter 6) argued at length for the undesirability of immortality. C.D. Broad, who like James, Schiller and Henri Bergson was one of several philosophically distinguished presidents of the Society for Psychical Research, famously concluded his assessment of the empirical indications for post-mortem survival by stating that he should be ‘slightly more annoyed than surprised’ to find himself surviving bodily death (Broad, 1962, p. 430).

A more general confession of a will to disbelieve was made by another eminent philosopher, Thomas Nagel:

Even without God, the idea of a natural sympathy between the deepest truths of nature and the deepest layers of the human mind, which can be exploited to allow gradual development of a truer and truer conception of reality, makes us more *at home* in the universe than is secularly comfortable. The thought that the relation between mind and the world is something fundamental makes many people in this day and age nervous. I believe this is one manifestation of a fear of religion which has large and often pernicious consequences for modern intellectual life (Nagel, 1997, p. 130, original italics).^7^

Finally, Hilary Putnam was comparatively vague when he stated that ‘‘Naturalism,’ I believe, is often driven by fear, fear that accepting conceptual pluralism will let in the ‘occult,’ the ‘supernatural’’ (Putnam, 2004, p. 66).

For what it’s worth, personally I find myself rather torn on the question whether magic and immortality are desirable. In my more introspective moments, I find Neoplatonic notions of a hidden interconnectedness of all living beings appealing, comforting and perhaps even conducive to mobilizing whatever little altruistic potential I might possess. On the other hand, the notion of other minds – incarnate as well as possibly discarnate – accidentally or intentionally snooping in the most intimate corners of my self, and having the power of manipulating and harming me through mere intentions, provokes a strong reaction of defence and unwillingness to grant the very possibility of transcendental correspondences. On a perhaps even more fundamental level, a part of me undoubtedly craves the kinds of social, aesthetic and intellectual fulfilments that life occasionally has to offer to continue indefinitely. But there are also moments when the prospect of a hypothetical impotence to end my existence if I wished so fills me with a feeling nothing short of a claustrophobic panic episode.

## Conclusion

The study of the ‘night side’ of nature may induce a sense of wonder, but it is also inevitably appended with a whole range of fundamental fears – in addition to the above, we could adduce the fear of being duped, of a loss of control, and not least the fear of ridicule. Historian Peter Lamont (2013) has criticized the continued lumping together of all sorts of deviant beliefs in modern psychological scales supposing to measure ‘paranormal belief’. There has been a wide spectrum of reasons for unorthodox beliefs over time, which psychologists are yet wont to ignore and sweepingly explain in terms of cognitive biases. With the psychology of paranormal belief continuing to thrive as a professional speciality, Lamont further notes a marked asymmetry in the complete absence of a tradition studying the psychology of paranormal *disbelief*. A similar asymmetry characterizes the public use of history in the continuing war against ‘superstition’, ‘irrationality’ and ‘pseudoscience’.

Immanuel Kant famously stated that the essence of Enlightenment thought was the abolishment of dogmatism and false authorities, supplanted by the cultivation of courage to think for ourselves, his motto being *sapere aude!* – dare to know! Kant’s appeal to intellectual courage necessarily admits fear. To radically think independently and question all authority is a scary thing indeed. But Kant himself did not follow his own principles when he responded to reports of ghostly goings-on with ridicule and armchair pathologization (Kant, 1900), an attitude that characterized the age of Enlightenment as much as undoubted advances in the cultivation of tolerance in other matters. The complimentary bogeys that plagued Kant and many of his contemporaries – the fear of materialism on the one hand, and of ‘enthusiasm’ (i.e. irrationality and ‘superstition’) on the other – continued throughout the nineteenth century and guided the professionalization of modern sciences.

The quasi-apocalyptic fears of supposed global dangers of magical belief that were so typical of the nineteenth century have not borne out, and in the face of recent historical studies documenting the integral role of continued occult mentalities in the making of modernity (cf. Albanese, 2007; Mannherz, 2012; Owen, 2004; Treitel, 2004), undiscriminating claims of a disenchantment of the world, let alone of the intrinsic backwardness and perilousness of occult beliefs, seem no longer feasible. But even though the original mentalities at work in the repudiation of radical empirical approaches to the occult may have vanished from public awareness, academic curricula still rest on epistemic prescriptions informed by these anxieties.

I might do worse than conclude these initial and somewhat crude observations with an appeal made by William James over a century ago: ‘We all, scientists and non-scientists, live on some inclined plane of credulity. The plane tips one way in one man, another way in another; and may he whose plane tips in no way be the first to cast a stone’ (James, 1897, p. 320). Some will no doubt misread this quote, along with my incomplete account of James’ pragmatist analysis of the psychology of belief above, as a call to a disastrous epistemic and scientific anarchism and relativism. But like James I prefer to say that a frank acknowledgement of the rationalist dilemma must not be confused with an excuse for lazy thinking and arrogant dogmatism. Far from paralysing our critical faculties, its admission might on the contrary motivate us to try harder than ever to identify, accept and eliminate inevitable biases standing in the way of our cultivating benevolent open-mindedness coupled with ‘never-sleeping suspicion of sources of error’ (James, 1897, p. 303).

## Notes on contributor

***Andreas Sommer*** is a historian of the human sciences with a background in philosophy, psychology and medical history. He is currently a Junior Research Fellow in History and Philosophy of Science at Churchill College and a teaching associate in the Department of History and Philosophy of Science at the University of Cambridge. Working on his first book, which reconstructs the co-emergence of psychical research and modern psychology in late-nineteenth century Europe and the US, he also hosts www.forbiddenhistories.com, a blog on the sciences and their historical links to the occult.

## Notes

1. Some new age writers have also twisted the history of science to fit their own agendas. For a critique, see Brooke and Cantor (1998, Chapter 3).

2. My translation.

3. Regarding public history, see, for example, the hair-raisingly biased Wikipedia entries on parapsychology and psychical research.

4. These have been documented en masse not only by unorthodox scientists but also by supposedly impartial historians and sociologists of science. For pertinent literature, see, for example, Sommer (2014a).

5. Regarding popular beliefs in the efficacy of prayer and healing intentions, Braude also remarks that ‘No process can be used only for the good. So, if we open the door to the salutary (or simply benign) effects of our thoughts on the external world, we must also open it to the destructive influence of our thoughts’ (loc. cit.).

6. While Grosso equates fears of the unknown with fears of the shadow in the Jungian sense, Jung himself resorted to anthropological arguments when he took issue with the ‘widespread bias’ against well-documented parapsychological phenomena, which to him revealed ‘all the symptoms of the primitive fear of ghosts’, for ‘even educated people who should know better occasionally utilize the most nonsensical arguments’, and may even ‘sign séance minutes and subsequently withdraw, as has been the case more than once, their signature, since what they had observed and verified was, as it were, impossible – as if one knew exactly what was possible!’ (Moser, 1950, p. 11, my translation).

7. On the question of theistic religion, Nagel continues: ‘I speak from experience, being strongly subject to this fear myself: I want atheism to be true and am made uneasy by the fact that some of the most intelligent and well-informed people believe in God and, naturally, hope that I’m right in my belief. It’s that I hope there is not God! I don’t want there to be a God; I don’t want the universe to be like that’ (loc. cit).
